# High Rates of Repeated Caesarean Section Deliveries and its Associated Maternal and Foetal Complications at A Tertiary Hospital in Northern Tanzania

**DOI:** 10.24248/eahrj.v8i1.754

**Published:** 2024-03-28

**Authors:** Anastazia J Ngao, Joseph Obure, Eusebious William Maro, Damian J Damian

**Affiliations:** a Kilimanjaro Christian Medical University College, Moshi, Tanzania; b Department of Obstetrics and Gynaecology, Kilimanjaro Christian Medical Centre, Moshi, Tanzania

## Abstract

**Background:**

About one-fifth of women undergo repeated caesarean section (RCS) deliveries worldwide. However, an increase in the number of RCS may lead to maternal and foetal morbidity and mortality. This study aimed to determine the rates of RCS deliveries and associated maternal and foetal complications at a tertiary hospital in northern Tanzania.

**Methods:**

This was a hospital-based cross-sectional study conducted at Kilimanjaro Christian Medical Centre (KCMC), Northern Tanzania. A total of 253 women who underwent caesarean section (CS) deliveries during the study period were included. Information from patient files was reviewed to abstract specific variables of interest, including maternal demographic and obstetric characteristics, maternal complications such as adhesions, postpartum haemorrhage, infections, anaesthetic complications, hysterectomy, and maternal deaths. Foetal complications related to RCS were also extracted, including the Apgar score, admission to the neonatal unit, neonatal infections, respiratory problems, and perinatal death.

**Result:**

A total of 253 women were enrolled in this study. Of these, 133 (52.5%) had RCS delivery. The mean (± standard deviation) age of women at enrolment was 29.9 (±6.5) years. The overall complications rate was 56.5% (32.9% among women having first CS and 67.1% RCS, *P<.001*). For women who underwent RCS, 37.2% had anaesthesia-related complications, including hypotension, nausea, bradycardia, difficult intubation, aspiration, and respiration. Other complications were sepsis (15%), postpartum haemorrhage (PPH) (11.9%), and wound dehiscence (5.5%). Only sepsis was independently associated with repeated CS delivery (adjusted odds ratio (aOR=11.3, 95% confidence interval [CI], 3.3 to 8.9; *P<.001*).

**Conclusion:**

The reported RCS in this study was high, associated with high CS complications. Necessary measures should be taken by healthcare providers to avoid unnecessary primary CS delivery, and counselling for trial of labour with close monitoring of labour for successful vaginal birth after caesarean section should be emphasised to avoid RCS and its complications.

## BACKGROUND

Caesarean section (CS) is among the most common obstetric procedures carried out worldwide. Globally, 18.6% of all births are estimated to be through CS, with repeated caesarean sections (RCS) increasing drastically, particularly in low-income countries.^1^ The World Health Organization (WHO) recommended a CS rate between 5 and 15% globally. However, higher CS rates have been reported in many countries worldwide.^2^ According to data from 169 countries with 98·4% of the world's births, estimated births that occurred through CS almost doubled from 2000 (16.0 million, 12.1%) to 2015 (29·7 million, 21.1%).^3^ Globally, there are regional and country-specific variations in CS rates, ranging from 58.1% in the Dominican Republic to as low as 0.6% in South Sudan.^3^ In Latin America and Caribbean regions, births by CS have been reported to be almost ten-fold higher (44.3%) than in the west and central Africa (4.1%). The in modern obstetricians and the number of facility deliveries have contributed to increased caesarean section rates worldwide.^3,4^ However, increasing CS delivery is associated with an increased risk of RCS in subsequent deliveries.

The rate of RCS deliveries is steadily increasing and is reported to vary across regions, ranging from 80% in high-income countries to 70% in low-income countries.^5^ In Sub-Saharan Africa, RCS has been reported to vary between counties and from public to private institutions. Studies from East Africa have reported that women who had prior CS deliveries were more likely to undergo RCS compared to their counterparts. In West Africa, a study involving 46 referral hospitals spread across Mali and Senegal reported RCS rates up to 38%.^6^ In South Africa, the rate of RCS ranged from 14% in public hospitals to 60% in private hospitals, whereas in Kenya, the rate of RCS has been reported to be relatively higher (67%) than what was recently reported in Tanzania (39.2%).^7,8^ A study in rural Tanzania reported that 10% of births were through CS, whereas 11% to 19% of births were RCS after the first CS and 9% to15% were RCS after two or more prior CS.^9^ A study done at a referral teaching hospital in Northern Tanzania reported an average CS rate of 33%, of which the RCS ranged from 17.1% to 26.8% between 2005 and 2010.^10^

Evidence from the literature has shown that RCS is associated with increased maternal and foetal complications. Women with RCS are at increased risk of having multiple intra-operative surgical complications, including adhesion, bleeding, an increased duration of surgery, bladder and bowel injuries, and wound sepsis.^11^ Moreover, other maternal complications linked to RCS include the development of placenta praevia, abruptio placenta, caesarean hysterectomy, and the need for blood transfusion.^12–15^ Foetal complications have also been associated with RCS, with inconsistent findings. While some studies have not associated RCS with perinatal complications^12–16^, some studies have reported preterm birth, an APGAR score below 7 in the 5^th^ minute, and neonatal ICU admission among women who have undergone RCS^14–16^ which are associated with increased subsequent caesarean sections.^4^

In Tanzania, there is limited data on the magnitude of RCS and its associated maternal and foetal complications. Available reports presented inconsistent results; for instance, a study done at Muhimbili National Hospital in Dar es Salaam reported a lower prevalence of maternal and foetal complications among women with previous CS delivery compared to those with previous vaginal delivery.^17^ This study aimed to determine the magnitude and complications associated with RCS in a tertiary teaching hospital in northern Tanzania.

## METHODS

### Study Design and Setting

This was a cross-sectional hospital-based study conducted from January to April 2018 at Kilimanjaro Christian Medical Centre (KCMC), a tertiary and teaching hospital located in Moshi, northern Tanzania. The hospital receives patients from the local community in the Kilimanjaro region as well as from the nearby regions, including Tanga, Arusha, and Manyara. KCMC has a catchment population of over 15 million people. About 3,885 women deliveries are reported annually, with caesarean section deliveries accounting for 43.5% of the deliveries.

### Study Population and Enrolment Procedures

All women who were delivered by CS at KCMC during the study period (from January to April 2018) were included in the study after they had signed informed consent. Three trained research assistants (midwives working with the obstetricians) administered face-to-face interviews through a structured questionnaire every morning (within 24 hours post-delivery) with all women who had been delivered by CS. Data were also extracted from the patients' files, and women were followed up for one week or until discharged from the hospital. The collected information included socio-demographic characteristics (age, marital status, education, occupation, referral type) and obstetric reproductive characteristics (parity, previous mode of delivery, gestational age, membrane status, history of stillbirth). Immediate maternal complications associated with RCS (anaesthesia, abnormal placentation, sepsis, post-partum haemorrhages, wound dehiscence, wound infection, bladder and bowel injury, endometritis, hysterectomy, and death). Information on foetal complications (hypoglycemia, neonatal infection, respiratory problems, foetal asphyxia, admission to the neonatal unit, early neonatal death) was also documented from the patients' files and from the neonatal unit registers.

### Statistical Analysis

The collected data were entered, cleaned, and analysed using Stata Version 16 (*Stata Statistical Software: Release 16*. College Station, TX: StataCorp LLC). A descriptive analysis was carried out to explore the socio-demographic, obstetric characteristics and foetal and maternal complications related to RCS. Numerical data were summarised using appropriate measures of central tendency and corresponding measures of dispersion, while categorical variables were summarised using frequency and percentage. Logistic regression analyses were used to determine the independent foetal and maternal complications related to RCS. A *P* value less than .05 was considered statistically significant in all tests.

### Ethics Approval and Consent to Participate

Ethical approval was sought from the Kilimanjaro Christian Medical College Research Ethics and Review Committee (CRERC-2230) of Tumaini University Makumira, while permission to conduct the study was granted by the Executive Director of KCMC Hospital. All women with one or more previous CS were explained about the study and given informed consent before data collection. Participants were assured of confidentiality and voluntary participation, and they were free to withdraw from the study at any point without affecting any medical care they deserved. The privacy and confidentiality of participants were carefully observed by using hospital numbers instead of participant names.

## RESULTS

### Sociodemographic Characteristics of the Participants

In total, 253 women who were delivered by CS were enrolled in this study, of which 133 (52.5%) had RCS. The mean (± SD) age of the participants at recruitment was 30 (± 6.5) years, ranging between 16 and 45 years. Most of the study participants were married (86.6%); had secondary education or above (65.2%); were self-employed (71.1%); multiparous (66.8%); had term gestational age (66.8%); had intact membrane status (73.1%), and they self-referred to the facility (64.4%). Compared to women having their first CS delivery, a significantly higher proportion of those having RCS delivery were older, multiparous, had a history of CS delivery, and had intact membrane status during the index delivery (Table 1).

**TABLE 1:** Sociodemographic Characteristics of the Participants by CS Delivery Status (N=253)

Characteristics	Total n (%)	Cesarean section status n (%)		*P-value*
		First	Repeated	
**Total**	**253**	**120 (47.4)**	**133 (52.6)**	
Age category (years)				
16–25	70 (27.7)	46 (38.3)	24 (18.1)	<.001
26–35	126 (49.8)	56 (46.7)	70 (52.6)	
36–45	57 (22.5)	18 (15.0)	39 (29.3)	
Marital status				
Married	219 (86.6)	101 (84.2)	118 (88.7)	.289
Unmarried	34 (13.4)	19 (15.8)	15 (11.3)	
Education level				
No formal	19 (7.5)	13 (10.8)	6 (4.5)	
Primary	69 (27.3)	33 (27.5)	36 (27.1)	.149
Secondary and above	165 (65.2)	74 (61.7)	91 (68.4)	
Occupation				
Employed	73 (28.9)	35 (29.2)	38 (28.6)	.917
Self-employed	180 (71.1)	85 (70.8)	95 (71.4)	
Parity				
1	84 (33.2)	75 (62.5)	9 (6.8)	<.001
2	71 (28.1)	26 (21.7)	45 (33.8)	
3	57 (22.5)	7 (5.8)	50 (37.6)	
4+	41 (16.2)	12 (10.0)	29 (21.8)	
Gestational age of the index delivery				
<37 weeks	84 (33.2)	45 (37.5)	39 (29.3)	.168
≥37 weeks	169 (66.8)	75 (62.5)	94 (70.7)	
Membrane status of the index delivery				
Intact	185 (73.1)	79 (65.8)	106 (79.7)	.013
Ruptured	68 (26.9)	41 (34.2)	27 (20.3)	
History of stillbirth				
Yes	35 (13.8)	16 (13.3)	19 (14.3)	.827
No	218 (26.9)	104 (86.7)	114 (85.7)	
Source of referral				
Institutional referral	90 (35.6)	46 (38.3)	44 (33.1)	.384
Self-referral	163 (64.4)	74 (61.7)	89 (66.9)	

### Maternal and Foetal Complications

**FIGURE 1:**
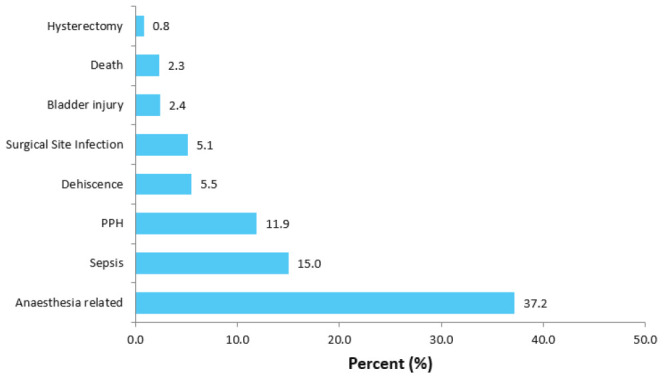
Maternal Complications of the Index Delivery (N=133)

Overall, more than half of participants (56.5%) had complications. Of these, 67.1% had RCS delivery. The most common reported or observed maternal complications were anaesthesia-related complications, i.e., hypotension, nausea, bradycardia, difficult intubation, aspiration, and respiration (37.2%), sepsis (15.0%), postpartum haemorrhage (11.9%), and dehiscence (5.5%) (Figure 1).

**FIGURE 2:**
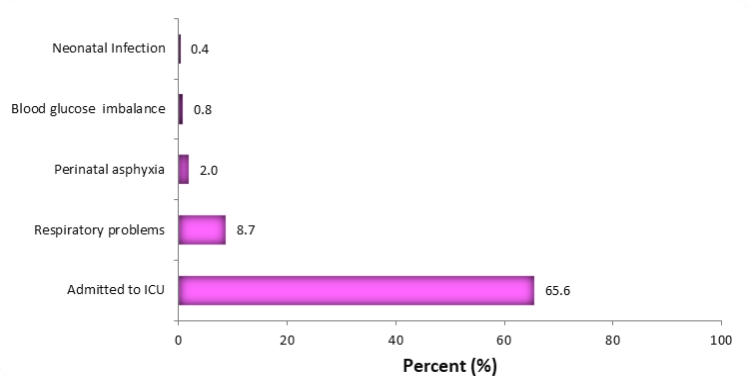
Foetal Complications of the Index Delivery (N=133)

The commonest foetal complication reported was admission to the neonatal intensive care unit (NICU) (65.6%). Other foetal complications included respiratory problems (8.7%), perinatal asphyxia (2.0%), hypoglycaemia, and neonatal infections (0.8% and 0.4%, respectively). (Figure 2).

### Maternal Complications Associated with RCS

Table 2 shows the maternal complications associated with RCS delivery. Only sepsis was independently associated with RCS delivery (aOR=11.3; 95% CI: 3.3 to 8.9; *P*<.001).

**TABLE 2:** Maternal Complications Associated with RCS Delivery (N=253)

Characteristics	Crude		Adjusted	
	OR¥ (95%CI)	*p-value*	aOR‡ (95%CI)	*p-value*
Anaesthetic complications				
No	Ref		Ref	
Yes	1.0 (0.6–1.6)	.914	1.0 (0.6–1.8)	.858
Sepsis				
No	Ref		Ref	
Yes	11.4 (3.4–38.3)	<.0001	11.3 (3.3–8.9)	<.0001
PPH				
No	Ref		Ref	
Yes	4.2 (1.6–10.6)	.003	2.6 (0.9–7.7)	.093
Dehiscence				
No	Ref		Ref	
Yes	5.9 (1.3–26.7)	.023	2.9 (0.5–6.2)	.222
Wound Infection				
No	Ref		Ref	
Yes	5.3 (1.2–24.5)	.032	4.2 (0.8–21.3)	.083
Bladder injury				
No	Ref		Ref	
Yes	1.8 (0.3–10.2)	.490	0.9 (0.1–7.8)	.915

‡ Adjusted odds ratio;

¥ Crude odds ratio

### Foetal Complications Associated with RCS Delivery

None of the perinatal factors were independently associated with RCS delivery.

## DISCUSSION

The result of this study showed that RCS contributes to more than half of all CS deliveries at KCMC and is significantly associated with a higher rate of CS complications compared to one CS delivery. The most common maternal complications associated with RCS were anaesthetic-related sepsis and postpartum haemorrhage, while neonatal ICU admission and respiratory problems were the most reported perinatal complications.

The high rate of RCS observed in this study is similar to that reported from low-income countries, in West and East African countries, including Tanzania.^5,6,8,16^ Studies in middle income countries like Ghana and most Arabic countries reported slightly lower RCS rates than those in our study.^18,19^ Contrary to our findings, a lower RCS rate has been reported in most developed countries.^20,21^ The reasons for the high rate of RCS in low-income countries include inappropriate counselling during antenatal visits and poor monitoring of labour, leading to unnecessary primary CS. Understaffing and poor resources in low-income countries have contributed to failure in labour trials after one CS delivery.^16^ Additionally, tertiary hospitals in low-income countries are influenced by the high number of obstetric emergency referrals, which can lead to a high number of CS. This is supported by another study conducted in Tanzania, which reported that over three quarters of all women with previous CS underwent RCS.^22^

Regarding maternal complications, this study found that half of mothers with RCS developed complications after delivery. Anaesthetic complications were found at higher rates than other complications. Other life-threatening maternal complications such as sepsis, postpartum haemorrhage, wound infections, dehiscence, and maternal deaths were also higher among mothers with RCS. This corroborates the findings from the study conducted in Turkey, which reported similar results.^23^ The high rate of complications due to RCS can be explained by the increased risk of pelvic adhesions and prolonged duration of surgery, which could both be attributed to both intra- and post-operative morbidities such as visceral injuries, severe haemorrhage, and severe post-operative infection.^24^ The increased incidence of morbidity among patients undergoing RCS was also reported to be attributed to the increased risk of placenta accrete, which is considered the leading cause of maternal morbidity and mortality in the US.^25^

The risk of perinatal complications associated with RCS has been described previously with inconclusive results. In Southeast Brazil, newborns from women who underwent CS delivery had a two-fold increase in neonatal near miss compared to neonates born form women with vaginal delivery.^26^ Conversely, Thailand reported higher rates of foetal complications among women with RCS although this association was marginally significant.^27^ In this study, perinatal complications including neonatal admission to intensive care unit, low Apgar score, and foetal death were higher among women with RCS. However, these were not significantly associated with RCS. The inconsistent findings from the current study underscore the need to conduct more studies with a larger sample size to clearly describe the actual impacts of RCS on foetal outcome and the pattern of foetal complications associated with it.

### Strength and limitations

The prospective nature of data collection from the study participants (interviews were done every morning within 24 hours post-delivery), eliminated the risk of recall bias and ensured accurate information was captured. Being a tertiary hospital, the study site serves more complicated cases and might result in higher rates of CS compared to other hospitals, which might affect the generalisability of the findings. In addition, information on the indication of primary CS was not obtained from many participants due to poor documentation in medical records.

## CONCLUSIONS

The proportion of repeated caesarean sections at KCMC is high, associated with high CS complications. In order to reduce the rates of RCS and the associated maternal and neonatal complications, health care providers should avoid unnecessary primary CS, provide proper counselling, and properly evaluate women who had one CS before performing an RCS to prevent maternal and neonatal complications.
